# Emergent phenotypic heterogeneity in synthetic cross-feeding consortia driven by heterogeneous metabolite distribution

**DOI:** 10.1093/nsr/nwag357

**Published:** 2026-06-12

**Authors:** Tianzheng Sun, Jing Xiao, Yuqian Zhang, Xijia Cao, Chenchao Xu, Kaihang Zhang, Shuyao Li, Lei Cheng

**Affiliations:** State Key Laboratory for Vegetation Structure, Function and Construction (VegLab), MOE Key Laboratory of Biosystems Homeostasis and Protection, College of Life Sciences, Zhejiang University, Hangzhou 310058, China; State Key Laboratory for Vegetation Structure, Function and Construction (VegLab), MOE Key Laboratory of Biosystems Homeostasis and Protection, College of Life Sciences, Zhejiang University, Hangzhou 310058, China; State Key Laboratory for Vegetation Structure, Function and Construction (VegLab), MOE Key Laboratory of Biosystems Homeostasis and Protection, College of Life Sciences, Zhejiang University, Hangzhou 310058, China; State Key Laboratory for Vegetation Structure, Function and Construction (VegLab), MOE Key Laboratory of Biosystems Homeostasis and Protection, College of Life Sciences, Zhejiang University, Hangzhou 310058, China; State Key Laboratory for Vegetation Structure, Function and Construction (VegLab), MOE Key Laboratory of Biosystems Homeostasis and Protection, College of Life Sciences, Zhejiang University, Hangzhou 310058, China; State Key Laboratory for Vegetation Structure, Function and Construction (VegLab), MOE Key Laboratory of Biosystems Homeostasis and Protection, College of Life Sciences, Zhejiang University, Hangzhou 310058, China; School of Life Sciences, Zhengzhou University, Zhengzhou 450001, China; State Key Laboratory for Vegetation Structure, Function and Construction (VegLab), MOE Key Laboratory of Biosystems Homeostasis and Protection, College of Life Sciences, Zhejiang University, Hangzhou 310058, China

**Keywords:** cross-feeding, cell noise, gene expression, phenotypic heterogeneity, synthetic microbial community

## Abstract

Cooperation is crucial for constructions of all levels of life. But cooperation is often vulnerable, and understanding the underlying causes remains a grand challenge in ecology and evolution. Here we, by constructing a synthetic cooperative system consisting of two genetically engineered *Escherichia coli* strains with metabolic cross-feeding, demonstrate that cross-feeding, though it promotes the growth, amplifies cell noise of both strain populations in the bacterial consortium. The emergent phenotypic heterogeneity, expressed as the induced increase in cell noise by cross-feeding, is attributed mainly to enhanced resource heterogeneity and subsequent variation in gene expression among cells within each population. We further show that the positive interaction (including mutualism, commensalism and/or exploitation) also induces phenotypic heterogeneity in distinct microbial consortia consisting of bacterial strains isolated from aquatic, soil or rhizospheric environments. Our results reveal a cooperation-induced trade-off between population fitness and homogeneity, highlighting the importance of stochasticity in understanding the evolution of cooperation in microbiomes in nature.

## INTRODUCTION

Life is built by cooperation [1,2]. Genes cooperate to produce cells. Cells cooperate to produce multicellular organisms. Individuals of multicellular or unicellular organisms rely on cooperation to form complex social populations and/or communities. In microbial communities, cross-feeding—the reciprocal exchange of excreted metabolites between different species or strains—represents a widespread and fundamental form of cooperation [3,4]. Cross-feeding can arise via several eco-evolutionary routes: from public goods secreted through leaky metabolism, to adaptive gene loss under the Black Queen Hypothesis and to selection for reciprocal traits [5,6]. Once established, cross-feeding can enhance population fitness by improving their resource use efficiency, increase growth rate through division of labor and/or reshaping metabolic networks and resist environmental disturbances (e.g. antibiotics) through synchronizing growth of different populations [4,7–9]. Nevertheless, cross-feeding is often vulnerable, and sometimes subject to collapse in the face of various environmental or evolutionary pressures [10–13]. Understanding the mechanisms underlying the maintenance and stability of cross-feeding in microbial communities is therefore a central question in ecology and evolution.

In theory, cooperative interactions can destabilize microbial communities because if one cooperator decreases in abundance it will likely pull down the abundance of other cooperators [10,14], and these changes have often been ascribed to external factors [15,16]. For example, in a synthetic microbial consortium consisting of two *Escherichia coli* mutant strains, the addition of amino acids (AAs) can cause a transition of interaction from positive to negative between two strains [17]. The introduction of a cheater who shares nutrient source with two cooperative *Lactococcus lactis* strains could outcompete the two cooperators, leading to the collapse of the cross-feeding system [18]. Recent studies have further shown that the emergence of non-cooperative phenotypes could alter the stability of cooperative populations or communities [19–21]. In a cross‑feeding consortium consisting of two *Pseudomonas stutzeri* strains that utilize naphthalene as a carbon source, some auxotrophic cells could spontaneously change to prototrophic phenotypes, enabling them to gain a fitness advantage under fluctuating environments, and thus ultimately disrupting metabolic collaboration between two strains [21]. Within a *Pseudomonas aeruginosa* population that displays cooperative expansion on a solid surface via the secretion of a biosurfactant (a public good), the occasionally arising phenotype that stops producing the biosurfactant (i.e. acting as a cheater) can outcompete cooperating cells because of low metabolic cost, thereby rendering the collapse of cooperation within the population [12]. These raise a question about whether metabolic exchange could affect stability in microbial cross-feeding systems through altering intrinsic fluctuation, or phenotypic heterogeneity, within microbial populations.

**Figure 1.**
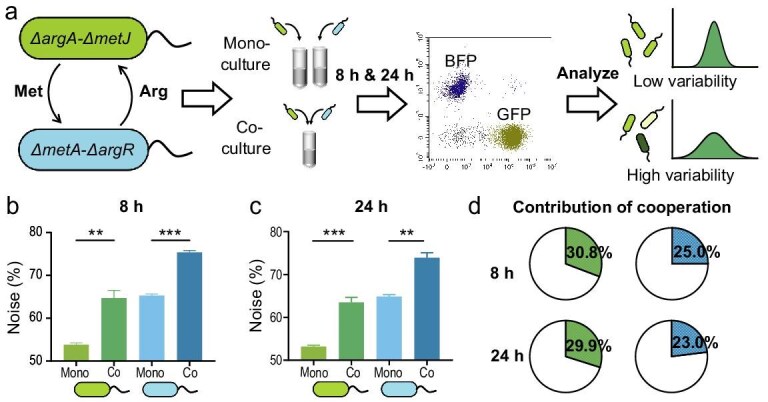
Cross-feeding increases cell noise in a synthetic microbial consortium. (a) Schematic description of community construction and noise detection. The two engineered strains, *ΔargA-ΔmetJ* (marked with a GFP) and *ΔmetA-ΔargR* (marked with a BFP), cooperated by exchanging two essential AAs (Met and Arg). The two strains were cultured in mono- or co-culture, with samples collected at 8 and 24 HAI for subsequent single-cell fluorescence intensity measurements using a flow cytometry. Cell noise was expressed using variability in fluorescence intensity. (b, c) Cell noise of *ΔargA-ΔmetJ* (left two bars) and *ΔmetA-ΔargR* (right two bars) populations under mono- or co-culture conditions at 8 (b) and 24 (c) HAI. Data represent mean ± SEM (standard error of the mean) (*n* = 3). ** and *** denote statistical significance at *P* < 0.01 and *P* < 0.001, respectively (two-tailed *t*-test). (d) Relative contribution of cross-feeding to cell noise of populations in the synthetic community. Left pies, *ΔargA-ΔmetJ*. Right pies, *ΔmetA-ΔargR*.

Cells, even genetically identical and subject to the same environmental and historical conditions, will display inevitable variations in form and functioning within a microbial population [22,23]. Such variability, termed also as phenotypic heterogeneity, may stem in large part from variation, or noise, in gene expression among cells [24–26]. However, it is hard to detect and differentiate cell noise from a plethora of biotic and abiotic factors since microbial communities in nature are very complex. To overcome this obstacle, we constructed a metabolic cross-feeding system consisting of two genetically engineered double-mutant strains of *E. coli, ΔargA-ΔmetJ* and *ΔmetA-ΔargR* [17,27,28]. *ΔargA-ΔmetJ* required arginine (Arg) to grow but overproduced methionine (Met), while *ΔmetA-ΔargR* needed Met but overproduced Arg (Fig. 1a), forming an obligate mutualistic relationship through exchanging essential AAs with each other (two-way positive interaction, +/+; Fig. S1a–d). We tracked the changes of cell noise by introducing green fluorescent protein (GFP) and blue fluorescent protein (BFP), encoded on plasmid pET-3a under the control of a lac promoter [29] (Fig. S1e–h), into *ΔargA-ΔmetJ* and *ΔmetA-ΔargR*, respectively. Cell noise ($\eta $) for each population ($\eta = \ \frac{\sigma }{\mu }$; where *σ* and *μ* are the standard deviation and mean of fluorescence intensity of sampled cells) was then determined by measuring single-cell fluorescent levels of the corresponding fluorescent protein using flow cytometry [25] (Fig. 1a).

## RESULTS

### Amplification of cell noise by cross-feeding

To investigate the influence of metabolic exchange on cell noise of the populations within a microbial community, we used the engineered cross-feeding system with different *E. coli* auxotrophic pairs to conduct three parallel studies within each of which overproducer and auxotroph were grown in either mono-culture or co-culture (Experiment 1). In the synthetic consortium consisting of *ΔargA-ΔmetJ* and *ΔmetA-ΔargR*, cross-feeding consistently increased cell noise of two populations for all six observations over the 48-h incubation period (Fig. S2a and b). At 8 and 24 hours after incubation (HAI) which corresponded to the logarithmic and stationary phases of cell growth (Fig. S1), particularly, both strains displayed substantially higher cell noise in co-culture compared to those in mono-culture (for *ΔargA-ΔmetJ, P* = 0.004 at 8 h, *P* = 0.001 at 24 h; for *ΔmetA-ΔargR, P* < 0.001 at 8 h, *P* = 0.002 at 24 h; Fig. 1b and c). By partitioning cell noise of each population in the community, cross-feeding contributed on average 30% and 25% of cell noise, respectively, for *ΔargA-ΔmetJ* and *ΔmetA-ΔargR* populations across the two time points within the synthetic consortium (Fig. 1d). Moreover, the cross-feeding-induced increase in cell noise was observed in two additional synthetic consortia consisting of either the *ΔargA-ΔmetJ* and *ΔmetA-ΔargR* strains with exchanged fluorescent proteins (Fig. S2e–h) or the other paired strains *ΔargA-ΔtrpR* and *ΔtrpC-ΔargR* (Fig. S2i–l). By plotting the standard deviation (*σ*) against the mean (*μ*) of population fluorescence intensity under both mono-culture and co-culture conditions, we also found that the observed increase in noise (*η*) is a consequence of disproportionate changes in *σ* relative to *μ* (Fig. S2c and d).

To determine whether the observed increase in cell noise could be generalized to microbial communities with positive interactions, we further constructed two synthetic consortia consisting of the *ΔargA-ΔmetJ* and *ΔmetA-ΔargR* strains in which either Arg or Met was added to form a commensalistic relationship (one-way positive interaction, +/0; Fig. S2m and o). For both two synthetic consortia with commensalism, only the auxotroph receiving nutritional benefit from an overproducer displayed an increase in population cell noise, while cell noise of the overproducer that did not obtain benefit from the other strain remained unchanged (Fig. S2n and p). Together, these results reveal that an increase in population cell noise emerges from assembling populations into a microbial cross-feeding community.

### Changes in cell noise driven by nutrient heterogeneity

**Figure 2.**
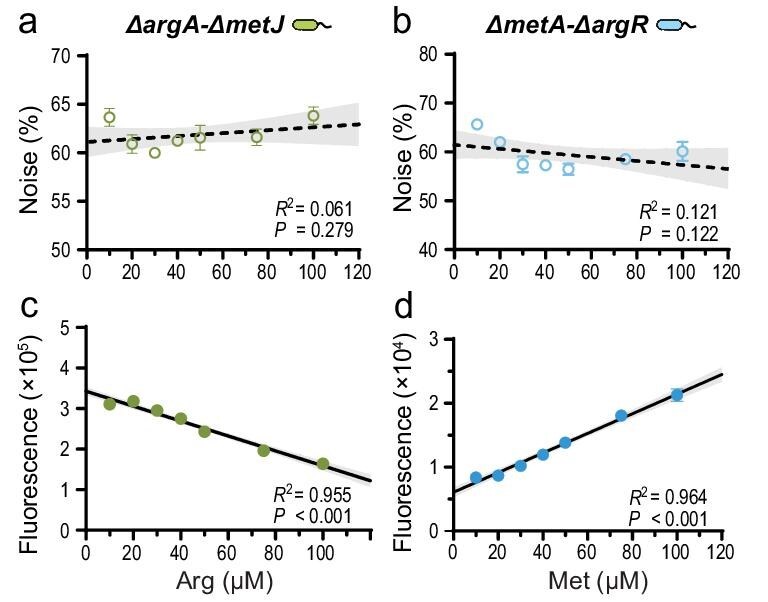
Cell noise and fluorescence intensity of the two cooperating strains supplied with a gradient concentration of AAs. The two engineered strains *ΔargA-ΔmetJ* marked with GFP (a, c) and *ΔmetA-ΔargR* marked with BFP (b, d) were incubated in mono-culture under different concentrations of Arg or Met, respectively. At 24 HAI, the average fluorescence intensity of GFP (c) or BFP (d) was measured and calculated from 10 000 single cells sampled from the corresponding population. Cell noise (a, b) was then calculated as the coefficient of variation (standard deviation/mean) of the fluorescence intensity. Dashed (a, b) and solid (c, d) lines represent linear regression fits, and the grey shaded areas indicate the 95% confidence intervals (CI). The slopes (*k*, mean ± 95% CI) are 0.015% ± 0.028% (a), −0.041% ± 0.053% (b), −1839 ± 193 (c) and 153 ± 14 (d), respectively. Data represent mean ± SEM (*n* = 3).

Because the concentrations of AA produced by metabolic exchange in co-cultures (15–25 µM) (Fig. S3a and b) differed substantially from those added to mono-cultures (e.g. 100 µM), we wondered whether the cross-feeding-induced increase in cell noise could be ascribed to the difference in nutrient availability between mono- and co-cultures. To test this hypothesis, we first grew *ΔargA-ΔmetJ* and *ΔmetA-ΔargR* under homogeneous conditions with Arg and Met, respectively, in mono-culture at the concentrations ranging from 10 to 100 µM (Experiment 2). As expected, increasing the supply of AA substantially promoted the growth of both *ΔargA-ΔmetJ* and *ΔmetA-ΔargR* (Fig. S3c and d). However, none of the two strain populations displayed any change in cell noise among different levels of AA addition (Fig. 2a and b; *P* = 0.279 for *ΔargA-ΔmetJ; P* = 0.122 for *ΔmetA-ΔargR*), neither did the same two strains with exchanged fluorescent proteins (Fig. S3k and l). Similar results were observed in a parallel culture study in which additional AA addition treatments at lower concentrations (e.g. 2–20 µM) were included in mono-cultures of both strains (Fig. S3g and h). Particularly, by comparing cell noise in mono-cultures of both strains between the AA addition treatments of 100 µM and the one close to the equivalent concentration measured in co-cultures (20 µM for Arg, and 10 µM for Met), no significant difference was detected for each of two strains (Fig. S3e and f). The observed unchanged cell noise among different levels of AA addition was likely due to the synchronizing changes between the standard deviation (*σ*) and mean (*μ*) of fluorescence intensity with increasing levels of AA supply (Fig. S3i and j).

Interestingly, the fluorescence intensity of sampled cells strongly correlated with the concentrations of supplied AA (*ΔargA-ΔmetJ, R*^2^ = 0.955, *P* < 0.001; *ΔmetA-ΔargR, R*^2^ = 0.964, *P* < 0.001; Fig. 2c and 2d). Such a strong correlation was also maintained in the same two strains carrying the exchanged fluorescent proteins (Fig. S3m and n), and its pattern could be influenced, at least in part, by codon composition and/or protein‑specific properties (Table S1 and Supplementary Note I) [30,31]. Together, these results suggest that nutrient (i.e. AA) availability governed single-cell gene expression levels, raising the possibility that cell noise amplification by cross-feeding may result from the intercellular variation in AA concentrations in our synthetic microbial systems.

**Figure 3.**
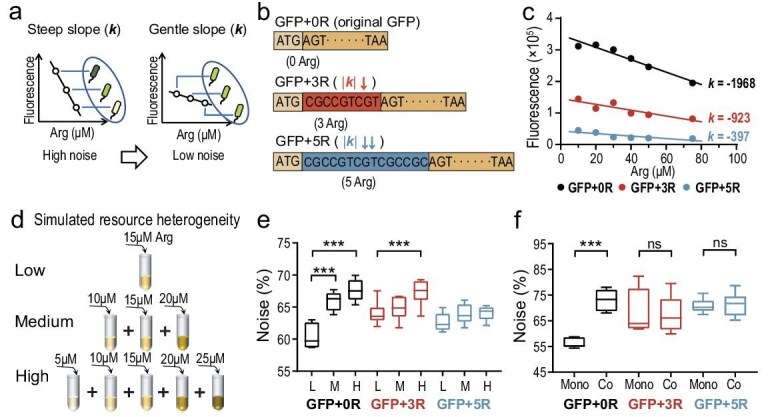
Amplification of cell noise driven by nutrient heterogeneity in the cooperative consortia. (a) Schematic depiction of the influence of nutrient (e.g. Arg) heterogeneity on cell noise, where the slope |*k*| represents the linear relationship between single-cell fluorescent intensities and AA concentrations. Strains with smaller |*k*| exhibit a lower cell noise. (b) Engineering three GFP mutant strains expected with different slopes. Strain *ΔargA-ΔmetJ* was used by inserting 0 (GFP + 0R), 3 (GFP + 3R) or 5 (GFP + 5R) additional Arg codon sequences after the start codon of GFP (ATG). (c) Obtained slopes for each of GFP mutant strains with distinct Arg codons. As the number of Arg codons increased, average single-cell fluorescence intensities responded less to increasing AA concentrations, as indicated by a gentler slope. The linear regression slopes (mean ± 95% CI) are −1968 ± 212 (*R*^2^ = 0.913), −923 ± 182 (*R*^2^ = 0.353) and −397 ± 90 (*R*^2^ = 0.702) for GFP + 0R, GFP + 3R and GFP + 5R, respectively (*P* < 0.001 for all). (d) Schematic diagram of three levels of nutrient heterogeneity: low (15 µM Arg), medium (equal mix of 10, 15 and 20 µM) and high (equal mix of 5–25 µM). (e) Effect of nutrient heterogeneity on cell noise of GFP + 0R, GFP + 3R and GFP + 5R. ‘L’, ‘M’ and ‘H’ represent low, medium and high levels of nutrient heterogeneity. (f) Cell noise of GFP + 0R, GFP + 3R and GFP + 5R strains in mono- or co-culture. In dot plot (c), data represent mean ± SEM (*n* = 6). In box plots (e, f), the center line represents the median, the box bounds represent the interquartile range (25th to 75th percentiles), and the whiskers extend to the minimum and maximum values (*n* = 6). ns, not significant. *** denotes significance at *P* < 0.001 (two-tailed *t*-test).

We therefore proposed that the heterogeneous distribution, rather than the overall absolute quantity, of metabolic nutrients (i.e. AA) would shape population-level gene expression variation or direct the changes of cell noise within microbial populations (Fig. S4). If this hypothesis holds, the slope (*k*) describing the changes of fluorescence intensity in response to the availability of AA should represent the extent to which nutrient heterogeneity affects cell noise. That is, the gentler the slope *k* is, the smaller influence of intercellular variation in the concentrations of AA on cell noise will be (Fig. 3a). Given that the slope *k* is an inherent characteristic of a gene or strain in response to nutrient availability, we engineered three mutants of *ΔargA-ΔmetJ* by adding 0, 3 or 5 Arg codons to the original GFP sequence, denoted as GFP + 0R, GFP + 3R and GFP + 5R, respectively (Fig. 3b). Increasing the number of Arg codons resulted in an increase in the percentage of Arg within the engineered GFP (Table S1), likely weakening the sensitivity of GFP gene expression in response to changes in the concentration of externally supplied Arg. This was verified by a culture study in which each of three strains was grown with the supply of a gradient concentration of Arg, with the one (i.e. GFP + 5R) containing more additional number of Arg codons having a gentler slope or smaller |*k*| value (Fig. 3c).

We then assessed the effect of nutrient heterogeneity on cell noise by growing each of GFP + 0R, GFP + 3R and GFP + 5R strains in mono-culture under different levels of nutrient heterogeneity (Experiment 3). Three nutrient heterogeneity levels were created by selectively combining mono-cultures pre-incubated with the supply of Arg at 5, 10, 15, 20 or 25 µM, homogeneously. The three levels were made at an equal final concentration of Arg (15 µM), with the low level only including the strain pre-incubated at 15 µM Arg while the medium and high levels being achieved by combining three (10, 15 and 20 µM) and five (5, 10, 15, 20 and 25 µM) pre-cultures (Fig. 3d). Increasing the levels of nutrient heterogeneity enhanced cell noise, particularly for the GFP + 0R strain (Fig. 3e). However, the magnitude effect of nutrient heterogeneity tended to decline for strains (e.g. GFP + 5R) with more additional Arg codons or lower |*k*| values (Fig. 3c and e), providing experimental evidence that linked gene expression variation, or noise, to nutrient heterogeneity among cells of the microbial populations.

Had nutrient heterogeneity been responsible for the cross-feeding-induced enhancement in cell noise, we would expect a lower or even neutral cross-feeding effect on cell noise in the synthetic consortia including GFP + 3R or GFP + 5R in comparison with GFP + 0R. To test this hypothesis, we grew each of the three strains, in combination with the cooperative strain *ΔmetA-ΔargR*, in mono-culture or co-culture (Experiment 4). As expected, we only observed a significant enhancement of cell noise in the co-culture for GFP + 0R, but not for GFP + 3R and GFP + 5R, compared to the corresponding population in the mono-culture (Fig. 3f; also see cell noise measurements for the strain *ΔmetA-ΔargR* in Fig. S5a). This Arg-dependent effect disappeared, when GFP with either 3 or 5 Arg codons was transferred into the Arg overproducer strain *ΔmetA-ΔargR* and co-cultured with the Arg auxotrophic strain *ΔargA-ΔmetJ*, manifesting as a consistent increase in cellular noise (Fig. S5b). Together, these results demonstrate that cross-feeding amplified population cell noise in the synthetic microbial consortia through increasing variation in gene expression in response to nutrient heterogeneity.

### Amplified cell noise under unevenly distributed strains

**Figure 4.**
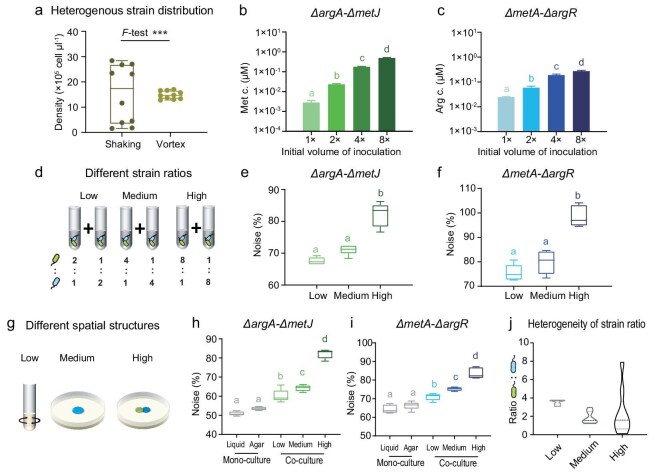
Enhancement of cell noise under different levels of strain distribution and spatial heterogeneity in the cooperative consortia. (a) Heterogeneous distribution of strains in the culture tubes. Each dot represents an individual sampling point from either the edge or center of the culture tube under two conditions: shaking at 200 r/min (left) or mixing with thorough vortex (right). The initial bacterial density was set at OD (optical density at 600 nm) = 1. Significance test on the difference of the variance of strain abundance between two treatments was performed by an *F*-test (***, *P* < 0.001). (b, c) LC–MS (liquid chromatography-mass spectrometry) quantification of AAs secreted by strain *ΔargA-ΔmetJ* (b) and *ΔmetA-ΔargR* (c) at 8 HAI under three inoculum densities (1× ≈ 2 × 10^4^ cells/μl). (d) Schematic diagram of three levels of strain distribution heterogeneity generated by mixing equal volume of pre-cultures with the *ΔargA-ΔmetJ*:*ΔmetA-ΔargR* ratios of 1:2 and 2:1 (low), 1:4 and 4:1 (medium) or 1:8 and 8:1 (high). (e, f) Effect of strain distribution heterogeneity on cell noise of *ΔargA-ΔmetJ* (e) and *ΔmetA-ΔargR* (f). (g) Schematic diagram of three levels of spatial heterogeneity. Low, liquid culture with shaking. Medium, two populations well mixed on plate. High, two populations placed adjacently but not mixed on plate. (h, i) Effect of spatial heterogeneity on cell noise of *ΔargA-ΔmetJ* (h) and *ΔmetA-ΔargR* (i) under mono- or co-culture conditions. (j) Variation of strain ratios under different levels of spatial heterogeneity. In bar plots (b, c), data represent mean ± SEM (*n* = 3). In box plots (a, e, f, h, i), the center line represents the median, the box bounds represent the interquartile range (25th to 75th percentiles), and the whiskers extend to the minimum and maximum values (a, *n* = 10; e and f, *n* = 6; h and i, *n* = 5). Individual data points are overlaid on panel a to show the distribution. In violin plot (j), the center line represents the median, the upper and lower lines denote the 25th and 75th percentiles, and the whiskers extend to the minimum and maximum values; the width of the violin reflects the local data density (*n* = 9). Different letters denote statistically significant differences (one-way ANOVA with Tukey’s post hoc test, *P* < 0.05).

In reality, nutrient heterogeneity within a metabolic cross-feeding system may arise, at least in part, from heterogeneous distribution of individual cells. To assess whether strain distribution heterogeneity exists within the synthetic consortia, we measured cell numbers at different positions (including the edge and the center) of the test tubes with three different initial cell densities under either shaking or vortex. Compared to the control group with vortex, variability in the strain abundance across different sampling points was substantially higher in the shaking tubes (Fig. 4a and Fig. S6a and b; *P* < 0.001, *F*-test), indicating that cells were unevenly distributed inside the tubes, even under shaking.

To further understand the rates of diffusion and uptake of AAs (Arg and Met) in a heterogeneously distributed cell system, we computed the Damköhler number (*Da*), defined as the ratio of macroscopic mixing time to reaction consumption time, using the continuous stirred tank reactor model (see Supplementary Note II). Our calculation, based on the concentrations of AA equivalent to the cross-feeding condition (20 µM) and free AA measured in the bulk solution of co-culture (mostly <1 µM at 24 h; Table S2), shows that *Da* was 19 in our shaking media. That is, the uptake rate of AA was ∼19-fold higher compared to the diffusion rate. The high Damköhler number, together with the low bulk AA concentrations, suggests that AAs secreted by an overproducer may be rapidly consumed by neighboring auxotrophic cells before they can diffuse farther, giving rise to resource heterogeneity induced by uneven distribution of cells. Along with the observation that the production of both Met (Fig. 4b) and Arg (Fig. 4c) in mono-cultures differed substantially among those with distinct initial inoculation cell densities, these results build a close link between uneven strain distribution and nutrient heterogeneity in the synthetic cross-feeding consortia.

To directly examine whether changes in the strain ratios could affect cell noise in the synthetic consortia with cross-feeding, we co-cultured *ΔargA-ΔmetJ* and *ΔmetA-ΔargR* under three different levels of heterogeneity in strain distribution (Experiment 5). The three levels were achieved by mixing equal volume of the pre-cultures with the *ΔargA-ΔmetJ*:*ΔmetA-ΔargR* ratios of 1:2 and 2:1 (low), 1:4 and 4:1 (medium) or 1:8 and 8:1 (high) (Fig. 4d). Increasing strain distribution heterogeneity elevated cell noise of both two populations within the synthetic consortia (Fig. 4e and f). Particularly, cell noise for *ΔargA-ΔmetJ* and *ΔmetA-ΔargR* was increased by 22% (*P* < 0.001, Fig. 4e) and 30% (*P* < 0.001, Fig. 4f), respectively, under the high level compared to the low level of strain distribution heterogeneity. In addition, temporal nutrient fluctuation, if existed due to heterogeneously distributed strains and/or nutrient diffusion over time, had a negligible role in regulating the changes in cell noise, as evidenced by results from a 12-h culture experiment (Fig. S6c) showing that there was no difference in cell noise for each of *ΔargA-ΔmetJ* (Fig. S6e) and *ΔmetA-ΔargR* (Fig. S6e) between one-shot and hourly additions of AA.

Microbial species and their individual cells are often spatially structured in nature, contributing to the heterogeneity of strain distribution within microbial communities. To determine whether spatial heterogeneity could play a role in shaping population cell noise, we designed an experiment in which *ΔargA-ΔmetJ* and *ΔmetA-ΔargR* were co-cultured in liquid cultures with shaking (low), in agar plates with two populations well pre-mixed (medium) or placed adjacently without pre-mixing (high) (Experiment 6, Fig. 4g). In mono-cultures, cell noise for both *ΔargA-ΔmetJ* and *ΔmetA-ΔargR* strains did not differ between liquid cultures and agar plates (Fig. 4h and i). In co-cultures, cell noise of each strain, no matter where they were grown, was higher than that in the corresponding mono-cultures, and its relative effect was strengthened with increasing levels of spatial heterogeneity (Fig. 4h and i). Also, we observed that variation in the ratios of *ΔargA-ΔmetJ* to *ΔmetA-ΔargR* became increasingly wide with elevating levels of spatial heterogeneity (Fig. 4j). These results suggest that amplification of cell noise by cross-feeding in spatially structured media was likely a result of enhanced heterogeneous distribution of individuals, and therefore nutrient heterogeneity surrounding them, in the synthetic microbial consortia.

### Increased cell noise in microbial consortia with positive interactions

**Figure 5.**
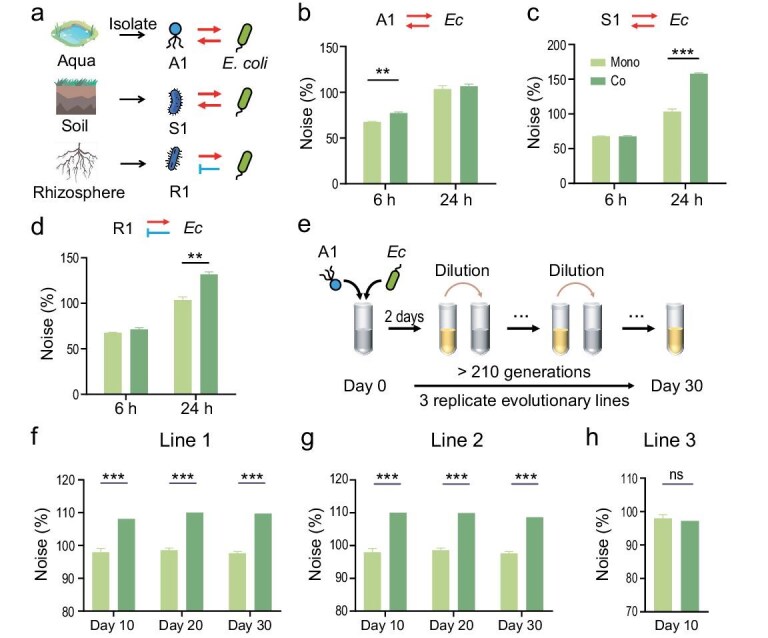
Changes of cell noise in the synthetic microbial consortia including strains from different environments. (a) Schematic diagram of three synthetic microbial consortia consisting of *E. coli*. and paired strain isolated from aquatic (A1), soil (S1) or rhizospheric (R1) environments. *E. coli* formed a cooperative (+/+, positive outcomes for both *E. coli* and A1/S1) relationship with either strain A1 or S1, but formed an exploitative relationship with R1 (+/−, positive outcome for *E. coli*, negative outcome for R1). The GFP-plasmid-carrying wild-type *E. coli* was used to monitor the changes of cell noise. Arrows, positive outcome; flat-headed arrows, negative outcome. (b–d) Cell noise of *E. coli* under mono- versus co-culture with A1 (b), S1 (c) and R1 (d) at 6 and 24 HAI. (e) Diagram of experimental design for a 30-day evolution of the *E. coli—*strain A1 consortium. The three evolutionary lines were maintained by serial passage every 2 days at a 1:40 dilution for a total of >210 generations. (f–h) Cell noise of *E. coli* for the three evolutionary lines measured on days 10, 20 and 30 under mono- and co-culture conditions. Data on line 3 (h) were only available on day 10, as it collapsed with the extinction of strain A1. All data represent mean ± SEM (*n* = 3), except for the co‑culture groups in the evolution experiment (f–h) in which each evolutionary line represents an independent replicate without biological replicates. ** and *** denote statistical significance at *P* < 0.01 and *P* < 0.001, respectively (two-tailed *t*-test, for f–h, using one-sample *t*-test).

To ascertain whether the change in cell noise induced by positive interactions could be applicable to communities in natural settings, we constructed three microbial consortia consisting of *E. coli* with strains A1, S1 or R1 which were isolated correspondingly from aquatic, soil and rhizospheric environments (Experiment 7, Fig. 5a). All three isolated strains exerted a positive effect on the growth of *E. coli* (Fig. 5a and Fig. S7a–c). Specifically, *E. coli* exhibited a cooperative or mutualistic relationship (+/+, positive outcomes for both *E. coli* and A1/S1) with both A1 and S1 (Fig. S7a and b), while formed an exploitative relationship with R1 (+/−, positive outcome for *E. coli*, negative outcome for R1), in which R1 facilitated the growth of *E. coli* but *E. coli* inhibited R1 slightly during the late incubation time (Fig. S7c). We then incubated each of the three isolated strains with the engineered *E. coli* to verify whether the positive interaction (either bidirectional or unidirectional) could lead to amplification of cell noise in *E. coli*. Consistent with previous observations (Fig. 1), cell noise of *E. coli* populations was in general increased within all the three microbial consortia over a 24-h incubation (Fig. 5b–d).

To further determine whether the observed increase in cell noise could be maintained over a relatively long time period, we performed a 30-day evolution study in which three evolutionary lines were established using the paired *E. coli* and strain A1 as a model cooperative system (Experiment 8, Fig. 5e). Over the whole experimental duration, which corresponded to >210 generations of *E. coli*, both evolutionary lines 1 and 2 had been sustained (Fig. S8a–i), with the increase of cell noise of *E. coli* populations being consistently observed on days 10, 20 and 30 (Fig. 5f and g). Interestingly, line 3 collapsed, as indicated by the observation of the extinction of strain A1 (Fig. S8a and j) accompanying with no change in population cell noise of *E. coli* on day 10 (Fig. 5h). Together, these results suggest that the increase of cell noise by positive interactions including cooperation may be widely occurred in microbial communities in various natural settings.

## DISCUSSION

This set of investigations demonstrates that cross-feeding amplifies cell noise of populations through increasing resource heterogeneity and subsequent variation in gene expression among cells of each population in microbial communities (Fig. S9). There has been a vast and rapidly growing body of data on the growth and dynamics of cooperators in microbial cross-feeding systems [10,32–35], but little attention has been paid to the intrinsic fluctuation, or noise, within populations of ecological communities, due partly to methodological limitations. This knowledge gap critically limits us from understanding and manipulating microbiomes for addressing environmental and health problems. Research into gene expression using isogenic cells has begun to uncover the sources of cell noise [25,26], but whether the inherently random nature of gene expression will be manifested in a complex population/community with intricate cell–cell interactions is poorly understood [36,37]. We, by constructing synthetic microbial consortia using strains with genetically identical background, revealed a trade-off mechanism in which metabolic cross-feeding, though stimulates the growth of strains, inevitably begets increased cell noise of two cooperative populations.

Our results that the observed increase in noise (*η*) reflects the altered *σ–μ* coupling (Fig. S2c and d) offer an insight into the source of population cell noise within the synthetic cross-feeding system. Because changes in *μ* are the result of altered cell state and growth stress [38,39], the *μ*-induced changes in cell noise primarily represent an extrinsic source; while changes in *σ* mainly reflect the stochasticity of gene expression process [24,40,41], the sources of *σ*-induced changes in noise should be considered both intrinsic and extrinsic. The observed disproportionate increase in *σ* relative to *μ* (Fig. S2c and d) therefore indicates that the cross-feeding-induced enhancement in cell noise may arise from both extrinsic and intrinsic noise components. Nevertheless, we suggest that future studies relying on dual-reporter systems [24] or monitoring the covariance of different gene expressions will help accurately differentiate intrinsic from extrinsic origins of population cell noise in microbial consortia with different types of interactions.

The finding of cross-feeding-induced enhancement of cell noise, typically manifested as variation in cellular phenotypes [22,42], suggests that cell–cell interactions may be an important factor governing the formation of phenotypic heterogeneity that has been widely observed for microorganisms. It has been shown that phenotypic heterogeneity could emerge as a consequence of metabolic specialization in relation to stress tolerance (i.e. heat or oxidative stress) [43]. A more recent study, by employing a yeast system consisting of plasmid-free auxotrophs and plasmid-carrying prototrophs, revealed that pyrimidine cross-feeding between individuals could promote cell-to-cell heterogeneity in plasmid-carrying populations under auxotrophic selection pressure [44]. Without imposition of any external biotic/abiotic stress or selection pressure, in the present study, we used the engineered cooperative bacterial system to reveal that metabolic exchange could promote phenotypic heterogeneity through amplifying cell noise within microbial populations sharing the same environmental background. Increasing phenotypic heterogeneity, on one hand, may promote population adaptation [22,45,46] and/or species coexistence [47] under fluctuating or stressful environments through switch of stochastic phenotypes or generation of multiple metabolic patterns. On the other hand, it may reduce the fitness of cell populations by pushing cells out of the optimal gene expression patterns [48] or help accumulate deleterious mutant phenotypes through restricting the efficiency of natural selection [49].

The emergent phenotypic heterogeneity in our cross-feeding system is mainly attributed to unevenly distributed metabolic nutrients. In a yeast fermenter with intense mixing, persistent microscale nutrient heterogeneity still exists because the Kolmogorov microscale is much larger than the diameter of yeast cells, limiting the mixing effect on nutrient distribution [50]. Similarly, the observed uneven spatial distribution of strains (Fig. 4a), in combination with the high Damköhler number (Supplementary Note II), in our incubation system with shaking, would inevitably create a heterogeneous metabolite distribution at microscopic scales. Spatial nutrient heterogeneity at microscopic scales has also been observed in natural aquatic systems [51]. For instance, by monitoring the microscale distribution of chemotactic bacteria, one study showed that nutrients released from the lysis of protozoa in the ocean diffuse radially [52]. Thus, the observed phenotypic heterogeneity driven by spatial nutrient heterogeneity in the present study may occur widespread in microbial populations in various natural settings. Moreover, previous work has suggested that nutrient limitation (e.g. via bet-hedging strategies) could also contribute phenotypic heterogeneity [53]. In our mono-cultures treated with a gradient of low AA concentrations (2–30 µM for both Arg and Met), the trend of cell noise changed slightly but not statistically significant with decreasing levels of AA (Fig. S3e–h), implying that nutrient limitation might play a role in facilitating the formation of phenotypic heterogeneity in our co-culture system. We suggest future studies with nuanced designs and high-resolution instrumentation to quantitatively differentiate the effect of nutrient limitation from spatial nutrient heterogeneity in the cross-feeding systems.

The discovery that cross-feeding amplifies cell noise highlights the role of stochasticity in understanding the ecology and evolution of microbial interactions in nature. First, it has been suggested that cross-feeding occurs because natural selection favors investment in cooperative rather than selfish behaviors. Our data suggest that the formation of stochastic phenotypes, likely driven by increased cell noise, could create serendipitous establishment of new niches with the possibility for the evolution of new cooperative relationships between individual cells within/between microbial populations [54–57].

Second, however, though cross-feeding benefits cooperators through providing essential nutrients and facilitating nutrient use efficiency [6], it may also jeopardize the stability of the cooperative relationship through increasing intrinsic noise of the whole system. For instance, the previously observed transition from positive (e.g. +/+, cooperation) to negative (i.e. −/−, competition) between two interacting cells/species [17,58] may be associated with the changes in cell noise, as cell populations under the competitive relationship tended to maintain unchanged or even at a lower cellular noise (Figs S7d–f and S10). This is probably because competition, though it could generate resource heterogeneity when species/strains compete for limiting resources, tends to select one particular efficient phenotype (e.g. being good at resource utilization and spatial expansion) in shared niches [59], resulting in reduced phenotypic heterogeneity [60]. The likelihood that the cooperative relationship will be collapsed in the face of increased uncertainty in gene functions exists as well [10,57,61,62]. Supporting evidence comes from the observation of the stochastic extinction of strain A1 in line 3 in the present work (Fig. S8j), though addressing the noise-mediated route to the breakdown of mutualism or cooperation will be a future research focus. Our results thus may also offer a possible compromise for the recent debate on whether and/or when positive interactions prevail in microbiomes in nature [4,63,64]. Finally, our findings suggest that there is a need to consider the changes of cell noise of populations induced by cell–cell interactions in future studies with the goal of rationally designing cooperation-predominated microbiomes to tackle environmental and health problems.

## MATERIALS AND METHODS

### Strains and plasmids

This study utilized four engineered *E. coli* K-12 MG1655 double-mutant strains, *ΔargA-ΔmetJ, ΔargA-ΔtrpR, ΔmetA-ΔargR* and *ΔtrpC-ΔargR* [17,65], containing different fluorescent reporter plasmids, pGFP, pBFP, pGFP + 3R and pGFP + 5R [29,66], to assemble synthetic cooperative consortia (also see Supplementary data). To mitigate potential plasmid loss during long-term laboratory evolution, a constitutive GFP reporter with T7 promoter was inserted into the *menF* locus in the genome of strain *E. coli* BL21(DE3), designated as BL21-GFP. Synthetic environmental communities were then assembled by pairing BL21-GFP with each of environmental isolates (three from Chaohu-lake water, two from Tianmu-mountain soil, one from Zhejiang-university maize rhizosphere).

All strains, plasmids and primers used in this study are listed in Tables S3 and S4.

### Culture conditions

*E. coli* strains were cultured aerobically in LB (Luria-Bertani broth) or M9-glucose media (M9 media) at 37°C, 200 rpm; communities containing natural isolates were cultured aerobically in TSB (tryptic soy broth) or M9 media at 30°C. Antibiotics, IPTG (Isopropyl β-D-1-thiogalactopyranoside) and AAs were added as required.

### Cultural experiments

Experiment 1: To investigate how cross-feeding affects gene expression noise, we constructed co-cultures of paired auxotrophic mutants, and monitored single-cell fluorescence to determine cell noise. Experiment 2: To determine the impact of AA concentration on cell noise, we grew mono-cultures of strains under a gradient of their required AA, respectively. Experiments 3–6: To test whether nutrient heterogeneity could alter noise, we experimentally manipulated low, medium and high levels of AA, strain ratio and spatial structure heterogeneity in synthetic consortia and conducted incubation experiments to measure cell noise, and also directly assessed the uneven distribution of strains within the culture system. Experiment 7: To examine whether positive interactions influence noise in real communities, we co-cultured *E. coli* with each of three beneficial isolates (from aquatic, soil and rhizosphere environments) and measured cell noise in *E. coli*. Experiment 8: To determine long-term changes in noise during co-evolution, we passaged co-cultures of *E. coli* and a beneficial strain A1 (*Priestia megaterium*) for >210 generations and periodically revived samples of the community to measure noise in the *E. coli* population.

Detailed information on Experiments 1–8 and other additional tests was presented in Supplementary data.

### Measurements

*Cell noise.* Cell noise (*η*) was quantified as the coefficient of variation (σ/μ) of single-cell fluorescence intensity, measured by flow cytometry with a minimum of 10 000 cells analyzed per sample.

*Quantitative real-time polymerase chain reaction (qPCR).* The absolute abundance of each engineered strain in a community was determined using qPCR that targeted unique genomic barcodes specific to each mutant (see primers in Table S4).

### Data analyses

Statistical analyses, including linear models, analysis of variance (ANOVA), TukeyHSD and *t*-tests, were performed using the R program (Version 4.3, The R foundation for Statistical Computing, 2023). The contribution of cross-feeding to total cell noise in the synthetic communities (Experiment 1) was computed by decomposing the total cell noise into noise inherent from the population and induced by cross-feeding ($\eta _{{\mathrm{comm}}}^2 = \eta _{{\mathrm{pop}}}^2 + \eta _{{\mathrm{cross}}\hbox{-}{\mathrm{feeding}}}^2$). The fold-changes of cell noise in Experiment 7 were calculated using the formula as follows: (${\mathrm{Noise\ change}} = (\eta _{{\mathrm{comm}}}- \eta _{{\mathrm{pop}}})/\eta _{{\mathrm{pop}}}\times 100\% $). Bootstrap methods were also applied to the calculations of the contribution of cross-feeding to total cell noise and the fold-changes of cell noise in Experiment 7 (also see Supplementary data). Statistical significance was accepted at *P* < 0.05.

More methods and materials are described in detail in the Supplementary data.

## Supplementary Material

nwag357_Supplemental_File
