# Supplementary material for: Emergent phenotypic heterogeneity in synthetic cross-feeding consortia driven by heterogeneous metabolite distribution
Source: Natl Sci Rev. 2026 Jun 12;13(15):nwag357. doi: 10.1093/nsr/nwag357 (PMC13463769; doi:10.1093/nsr/nwag357)
Supplement: Supplementary file 1 [file nwag357_supplemental_file.docx]

**Supplementary Information for**

**Emergent phenotypic heterogeneity in synthetic cross-feeding consortia driven by heterogeneous metabolite distribution**

Tianzheng Sun^1^, Jing Xiao^1^, Yuqian Zhang^1^, Xijia Cao^1^, Chenchao Xu^1^, Kaihang Zhang^1^, Shuyao Li^2,*^ and Lei Cheng^1,*^

^1^ State Key Laboratory for Vegetation Structure, Function and Construction, MOE Key Laboratory of Biosystems Homeostasis and Protection, College of Life Sciences, Zhejiang University, Hangzhou 310058, China

^2^ School of Life Sciences, Zhengzhou University, Zhengzhou 450001, China

* E-mails: [lcheng@zju.edu.cn](mailto:lcheng@zju.edu.cn), [shuyaoli1021@163.com](mailto:shuyaoli1021@163.com)

**Contents**

1. Detailed materials and methods
2. Supplementary notes
3. Supplementary Figures 1-10
4. Supplementary Tables 1-5
5. Cited References
1. **Detailed materials and methods**

**Strains and plasmids**

We engineered mutant strains from laboratory preserved wild-type strain *Escherichia coli* K-12 MG1655 by deleting certain genes from the genome, as previously described [1, 2]. Three auxotrophs (*ΔargA*, *ΔmetA* and *ΔtrpC*) of arginine, methionine and tryptophan were constructed by deleting genes *argA*, *metA* and *trpC* from wild type *E. coli*, respectively. Meanwhile, three overproducers (*ΔargR*, *ΔmetJ* and *ΔtrpR*) of the corresponding amino acids (AAs) were engineered by deleting genes *argR*, *metJ* and *trpR*, separately. On the basis of these auxotrophs and overproducers, we then engineered four double-mutant strains, *ΔargA-ΔmetJ*, *ΔargA-ΔtrpR*, *ΔmetA-ΔargR*, *ΔtrpC-ΔargR*, which shall be used to construct cooperative bacteria consortia.

We also engineered four plasmids containing genes encoding different fluorescence proteins. Each plasmid was constructed by inserting the corresponding fluorescence protein genes with a lac operator into the plasmid pET-3a [3] between the restriction sites BglII (agatct) and HindIII (aagctt). The lac operon comprises a promoter P_lac_ and an operator gene *lacO*, and the full transcriptional activity of lac operon can be induced by Isopropyl β-D-1-thiogalactopyranoside (IPTG). Two plasmids, pGFP and pBFP, were engineered to express enhanced green fluorescent protein (EGFP) gene and blue fluorescent protein (mTagBFP2) gene, respectively [4]. Plasmids pGFP+3R and pGFP+5R were engineered by introducing three and five arginine codons (CGC or CGT, see the sequence in Fig. 3b) to pGFP following translation initiation site (ATG) of the EGFP gene, respectively. These plasmids were constructed on the basis of standard cloning and DNA recombination techniques and verified by DNA sequencing using universal primers of pET3a.

Specifically, the reporter gene sequence contains an engineered lac promoter cassette and the fluorescent protein coding sequence. The lac promoter cassette includes the promoter (33 bp: 52-84), operator (21 bp: 91-111), and ribosome binding site (5 bp: 118-122), followed by the fluorescent protein sequence (from “atg” to “taa”). The sequence is formatted as follows: the promoter underlined, the operator in italics, the ribosome binding site in bold; the restriction enzyme sites, start codon, and stop codon of the fluorescent protein in lowercase.

**Lac+EGFP：**

agatctGCGCAACGCAATTAATGTGAGTTAGCTCACTCATTAGGCACCCCAGGCTTTACACTTTATGCTTCCGGCTCGTATGTTGTGTGG*AATTGTGAGCGGATAACAATT*TCACACAG**GAAACAG**CTatgAGTAAAGGAGAAGAACTTTTCACTGGAGTTGTCCCAATTCTTGTTGAATTAGATGGTGATGTTAATGGGCACAAATTTTCTGTCAGTGGAGAGGGTGAAGGTGATGCAACATACGGAAAACTTACCCTTAAATTTATTTGCACTACTGGAAAACTACCTGTTCCATGGCCAACACTTGTCACTACTTTCGGTTATGGTGTTCAATGCTTTGCGAGATACCCAGATCACATGAAACAGCATGACTTTTTCAAGAGTGCCATGCCTGAAGGTTATGTACAGGAAAGAACTATATTTTTCAAAGATGACGGGAACTACAAGACACGTGCTGAAGTCAAGTTTGAAGGTGATACCCTTGTTAATAGAATCGAGTTAAAAGGTATTGATTTTAAAGAAGATGGAAACATTCTTGGACACAAATTGGAATACAACTATAACTCACACAATGTATACATCATGGCAGACAAACAAAAGAATGGAATCAAAGTTAACTTCAAAATTAGACACAACATTGAAGATGGAAGCGTTCAACTAGCAGACCATTATCAACAAAATACTCCAATTGGCGATGGCCCTGTCCTTTTACCAGACAACCATTACCTGTCCACACAATCTGCCCTTTCGAAAGATCCCAACGAAAAGAGAGACCACATGGTCCTTCTTGAGTTTGTAACAGCTGCTGGGATTACACATGGCATGGATGAACTATACAAAtaaaagctt

**Lac+mTagBFP2：**

agatctGCGCAACGCAATTAATGTGAGTTAGCTCACTCATTAGGCACCCCAGGCTTTACACTTTATGCTTCCGGCTCGTATGTTGTGTGG*AATTGTGAGCGGATAACAATT*TCACACAG**GAAACAG**CTatgGTTAGTAAGGGCGAGGAATTGATTAAGGAGAACATGCATATGAAGCTGTACATGGAAGGCACTGTGGATAACCACCACTTTAAATGCACCAGTGAAGGCGAAGGCAAGCCATATGAGGGAACACAGACTATGCGGATTAAAGTGGTGGAGGGTGGCCCATTGCCATTCGCTTTTGACATTCTCGCTACCAGTTTTCTTTATGGGTCCAAGACCTTCATAAATCATACTCAGGGGATCCCAGACTTCTTCAAACAATCTTTTCCGGAAGGGTTCACCTGGGAGCGGGTGACAACTTACGAGGATGGGGGCGTGCTGACCGCAACTCAGGATACCAGCCTCCAGGACGGATGCTTGATCTATAACGTTAAGATCCGCGGCGTGAATTTTACCTCAAATGGGCCGGTGATGCAGAAAAAGACCCTGGGATGGGAGGCTTTCACTGAAACGCTCTACCCCGCTGATGGCGGCCTGGAGGGCAGAAATGATATGGCCCTGAAGCTGGTGGGAGGGAGCCACCTTATAGCTAACGCCAAGACCACTTACCGCTCCAAAAAGCCAGCAAAGAATCTCAAGATGCCCGGGGTGTACTACGTTGATTACCGCCTGGAGAGGATCAAGGAAGCCAATAACGAGACCTACGTCGAGCAGCACGAGGTGGCCGTTGCCCGGTACTGCGATCTTCCAAGTAAACTTGGCCATAAGCTGAATtaaaagctt

To mitigate potential plasmid loss during the long-term laboratory evolution experiments (Experiments 7 and 8), we constructed a chromosomally integrated reporter strain. A constitutively expressed gfp cassette under the control of the T7 promoter was inserted into the *menF* locus of our laboratory stock *E. coli* BL21(DE3), yielding the strain designated BL21-GFP. Synthetic microbial communities were then assembled by pairing BL21-GFP with each of environmental isolates, and cell noise was quantified by monitoring the inter-cellular variability of GFP fluorescence within *E. coli* populations. The T7 promoter sequence is as follows (the promoter underlined and the ribosome binding site in bold):

TAATACGACTCACTATAGGGAGACCACAACGGTTTCCCtctagaAATAATTTTGTTTAACTTTAAG**AAGGAG**ATATA. The gfp sequence is identical to that used in the Lac‑driven GFP reporter.

To construct microbial consortia using strains from natural settings, we isolated six environmental strains from aquatic, soil, and rhizospheric environments. Specifically, water samples were collected from the shoreline of Chaohu Lake, in Anhui province in China. Soil samples were collected from a subtropic forest at Tianmu Mountain, in Zhejiang province with an elevation of 800 m. Plant rhizospheric samples were obtained from maize plants grown at the Agricultural Experiment Station of Zhejiang University. Prior to experimentation, all isolates were pre-cultured in Tryptic Soy Broth (TSB), a nutrient-rich medium, to ensure robust growth.

All strains, plasmids and primers used in this study are listed in Tables S3 and S4.

**Culture conditions**

*E. coli* strains were cultured aerobically in LB or M9-glucose minimal media (M9 media) at 37°C. Environment isolates were cultured aerobically in TSB or M9 media at 30°C. Liquid cultures were incubated with shaking at 200 r.p.m., and M9 agar media were incubated in a thermostatic incubator. The LB media included 10 g/L tryptone, 10 g/L NaCl and 5 g/L yeast extract. The TSB media included 30 g/L Tryptic Soy Broth. The M9 media contained M9 salts (6.8 g/L Na_2_HPO_4_, 3 g/L KH_2_PO_4_, 1 g/L NH_4_Cl, 0.5 g/L NaCl), 0.493 g/L MgSO_4_, 0.011 g/L CaCl_2_ and 0.4% (wt/vol) glucose. Antibiotics (ampicillin, 100 μg/ml), Isopropyl β-d-1-thiogalactopyranoside (IPTG, 48 μg/ml) and amino acids (~ 0.1 mM) were added as required. For mix-cultures, no additional amino acids were supplied. For the M9 agar media, 15 g/L agar were added. Ampicillin, IPTG and amino acids were then added when the agar medium was cooled to nearly 60℃ to prevent it from degradation at high temperatures. All chemicals were purchased from Sigma-Aldrich Co. unless otherwise stated.

**Experiment 1**

We constructed the cooperative microbial consortia using paired mutant strains of either *ΔargA-ΔmetJ* and *ΔmetA-ΔargR*, or *ΔargA-ΔtrpR* and *ΔtrpC-ΔargR*. Each of these strains was pre-cultured overnight in LB media with selective antibiotics added appropriately. The resulting early-stationary-phase cells were washed twice and resuspended using the fresh M9 media. Cells were then harvested and used to construct experimental communities (co-cultures, 2 mL), each with a starting density of OD_600_ = 0.02 (OD_600_ denotes optical density at 600 nm measured using a spectrophotometer (Unico Instrument Co., Ltd., UV-2802)). For mono-cultures, required AA (Arg, Met or Trp; 0.1 mM) was added. Ampicillin and IPTG were also added if required. Three replicates were set for each treatment. Samples (20μL) were collected from each replicate at 4, 8, 12, 24, 32 and 48 hours after incubation (HAI). Samples were then used for determining single-cell fluorescence using a flow cytometry and subsequent cell noise quantification (see Measurements).

To determine the growth of strains under different culture conditions, samples were collected every 2h from inoculation till 24HAI. All samples were frozen at -20℃, and the abundance of each strain was determined using qPCR.

The issue of horizontal gene transfer was not considered in this work, as previous studies with the similar systems showed that neither reverse mutation nor plasmid transfer occurred during the 24h experimental time [1, 5].

Cell‑to‑cell variation in plasmid copy numbers might contribute to the change of cell noise. Nevertheless, our design that both mono- and co-culture shared the same plasmids ensures that it would not alter the effect of cooperation on population cell noise for each set of experiments. One previous study also showed that plasmid copy number fluctuations, even existed within a population, contribute little to the changes of overall noise [6].

**Experiment 2**

To test the impact of AA addition on cell noise, we grew *ΔargA-ΔmetJ* and *ΔmetA-ΔargR* in mono-culture under the supply of a gradient concentration of Arg and Met, respectively. Other incubation conditions were the same as those of the mono-cultures in experiment 1. The initial AA concentrations were set at 10 μM, 20 μM, 30 μM, 40 μM, 50 μM, 75 μM or 100 μM. Each treatment was repeated three times. Sampling for florescence intensity measurement and cell noise calculation was identical to that of in experiment 1. Data on single-cell fluorescence intensity and cell noise were fitted with linear models (95% confidence interval).

**Experiment 3**

To test whether nutrient heterogeneity alters cell noise, we mono-cultured three GPF mutant strains under three different levels of nutrient heterogeneity. Strain *ΔargA-ΔmetJ* (carrying one of the GFP plasmid, GFP+0R, GFP+3R or GFP+5R, see the Strain and plasmids) was pre-cultured under each of five different Arg concentrations (5 μM, 10 μM, 15 μM, 20 μM and 25 μM) over 24h. The three levels of nutrient heterogeneity were manipulated by mixing different combinations of pre-cultures at an equal concentration of AA. The low level was achieved by only taking the strain pre-incubated with 15 μM Arg, the medium level was achieved by equally mixing cultures pre-incubated with 10, 15 and 20 μM Arg and the high level was achieved by equally mixing cultures pre-incubated with 5, 10, 15, 20 and 25 μM Arg. Each group had six replicates. Sampling for cell noise calculation was identical to that of experiment 1.

**Experiment 4**

To directly investigate the effect of nutrient heterogeneity on cell noise under co-culture conditions, we constructed two sets of synthetic consortia with cross-feeding. In the first set, *ΔargAΔmetJ* expressing one of the three GFP mutants (GFP+0R, GFP+3R, or GFP+5R) was co‑cultured with *ΔmetAΔargR*-BFP. In the second one, *ΔmetAΔargR* expressing one of the three GFP mutants was co‑cultured with *ΔargAΔmetJ*-BFP. Incubation conditions and sampling for fluorescence intensity measurement were identical to that of experiment 1.

**Experiment 5**

To test whether changes in the ratio of two cooperative strains affect cell noise, we incubated the cooperative consortia consisting of *ΔargA-ΔmetJ* and *ΔmetA-ΔargR* under three different levels of strain distribution heterogeneity. Cocultures of *ΔargA-ΔmetJ* and *ΔmetA-ΔargR* were first pre-incubated with the initial inoculation ratios of *ΔargA-ΔmetJ* to *ΔmetA-ΔargR* at 8:1, 4:1, 2:1, 1:2, 1:4 or 1:8 over 24h. Each of the three levels of strain distribution heterogeneity was then manipulated through mixing pre-cultures with two different initial strain ratios at an equal volume. Specifically, the low, medium, and high levels were achieved by mixing the pre-cultures with the initial inoculation ratios of *ΔargA-ΔmetJ* to *ΔmetA-ΔargR* at 2:1 and 1:2, 4:1 and 1:4 and 8:1 and 1:8, respectively, simulating a situation in which a population with a macroscopic 1:1 strain ratio is divided into multiple patches, with patches differing in their local strain ratios. Each set had six replicates. Incubation conditions and sampling were identical to that of experiment 1.

**Experiment 6**

To test whether spatial structure heterogeneity influences cell noise, we co-cultured strains *ΔargA-ΔmetJ* and *ΔmetA-ΔargR* with three different levels of spatial heterogeneity. For the low level of spatial heterogeneity, strains *ΔargA-ΔmetJ* and *ΔmetA-ΔargR* were cultivated together in M9 liquid media with shaking to achieve a relatively homogeneous growth condition. Incubation of mono- and co-culture were identical to that of experiment 1. The medium and high levels of spatial heterogeneity were achieved by co-culturing strains in M9 agar media, with the mixture of the two strains at an equal volume of suspension cultures placed in the center of AA-free agar plate or the suspension cultures of the two strains at an equal volume dropped adjacently but with limited contact in the center of the AA-free agar plate. For mono-cultures, 20μL of M9-washed suspension culture was dropped in the center of agar plates with corresponding AAs added. Each treatment had six replicates. For strain ratio measurements, samples from nine randomly chosen points were taken from one of the six replicates of each treatment at 24 HAI. Samples from liquid media were taken by collecting 20μL bacterial fluid, whereas those from agar media were sampled using sterilized toothpicks from randomly selected locations. The rest of the other five replicates of each treatment, we rinsed the colonies off the plates softly with 1mL of fresh M9 media which was then used for single-cell fluorescence intensity measurements and subsequent cell noise calculations.

**Experiment 7**

To examine whether the positive interaction including mutualism influences cell noise in real microbial communities, we constructed three bacterial consortia consisting of *E. coli* (Bl21-GFP) and each of three bacterial strains isolated from different environments. The three bacterial strains were isolated from aquatic, soil and plant rhizospheric environments, labelled as A1, S1 and R1, respectively, and each of them facilitated the growth of *E. coli* (Table S3). We co-cultured *E. coli* and each of A1, S1 and R1 strains in M9 liquid media for 24 hours. The incubation was similar to that of experiment 1, except that strains were pre-cultured overnight in TSB media. Each treatment had three replicates. Samples of mono- and co-culture of each consortium were taken at 6, 12 and 24 HAI to measure the single-cell fluorescence intensity of *E. coli* and to monitor the abundance of both *E. coli* and isolated strains. In the current study, we did not monitor cell noise changes in populations of environmental strains, as it was a little bit of technically challenging and time‑consuming to introduce fluorescent protein‑encoding plasmids into these strains. Though it would be ideal for monitoring all species, current experimental setup was useful for understanding the effect of positive interactions on population cell noise in synthetic microbial consortia with paired species.

**Experiment 8**

To determine the relatively long-term changes of cell noise in microbial consortia with positive interactions (i.e. cooperation), we co-cultured strain A1 with *E. coli* (Bl21-GFP) over 30-day which corresponded to >210 generations of *E. coli* (the number of cell generations was estimated on the basis of a seminal long‑term evolution experiment (LTEE) with *E. coli* [7]). Both two strains were pre-cultured overnight in TSB media, with other initial culture conditions identical to that in Experiment 1. Two treatments were established: (i) *E. coli* evolution alone (for three replicates) and (ii) co-evolution of *E. coli* and strain A1 (three evolutionary lines, each of them representing an independent replicate). Cultures were passaged at a 1:40 dilution into fresh M9 medium every 48h. Samples were collected every 10 days and then stored at -20 ℃. All frozen samples were revived and cultured in M9 medium for single-cell fluorescence intensity measurements of *E. coli* populations.

**Strain distribution in culture tubes**

We performed all studies using culture tubes under shaking at 200 rpm, a typical method adopted in microbiological studies, especially for *E. coli* [8]. To test whether strain distribution is homogeneous in the culture tubes during incubation, we conducted a culture test by comparing strain distribution between shaking and vortex. The strain *ΔargAΔmetJ* was pre‑cultured overnight in LB media, and was then washed twice and cultured in M9 media with the initial cell density (OD_600_) set at 1, 0.6, or 0.3. After 4 hours of shaking incubation, samples were then collected immediately from the edge and center of the tubes under two conditions: shaking at 200 rpm or mixing by a thorough vortex. Each sampling position was replicated five times. After a quick sampling, the tubes were returned to the 200 rpm incubator (for shaking) or the vortexer (for vortex mixing) for the next possible sampling. The strain abundance of each sample was quantified by qPCR. The variance in strain abundances between shaking and vortex conditions (each consisting of 5 replicates × 2 positions = 10 samples) was compared using an *F*‑test.

**AA production in the metabolic cross-feeding system**

To determine the AA yields in the cross-feeding consortia, we mono-cultured strains *ΔargA-ΔmetJ* and *ΔmetA-ΔargR* under four initial inoculum volumes. The M9-washed suspension cultures of each strain were inoculated at initial optical densities (OD_600_) of 0.02 (denoted as 1×), 0.04 (2×), 0.08 (4×) and 0.16 (8×), respectively. At 8 HAI, the entire 2mL bacterial fluids were collected for amino acid concentration measurement.

**Effect of temporal fluctuation of AA supply**

To test the effect of temporal fluctuation in the intra-cellular AA concentration on cell noise, we designed a culture study by growing strains *ΔargA-ΔmetJ* and *ΔmetA-ΔargR* in mono-culture with the addition of AAs either on one-shot or an hourly basis. Each treatment had three replicates. For the one-shot nutrient addition treatment, AA was added at the onset of the experiment with a concentration of 30 µM, which was far more than required throughout the entire 12-hour cultivation despite gradual consumption, thereby supporting a steady-state nutrient supply. For the hourly addition treatment, AA was added at a rate of 2.5 µM per hour, resulting in temporal fluctuations of AA over the course of the experiment due to the continuous consumption and supply. At 130 minutes after incubation (namely, 10 minutes after AA addition), 20 µL samples were collected and then repeated every 2 hours.

**Measurements**

*Cell Noise* We defined cell noise of each strain population as the coefficient of variation (*η*) of single-cell fluorescence intensity, which is equal to the ratio of standard deviation to mean (*σ/μ*) [9, 10]. Single-cell fluorescence intensity was determined using a flow cytometry (Beckman Coulter, Inc., CytoFLEX). Bacterial cells expressing green fluorescent protein (GFP) were detected on the FITC channel using 488 nm excitation light, and bacterial cells expressing blue fluorescent protein (BFP) were detected on the PB450 channel using 405 nm excitation light. After cultivation, bacterial samples were diluted to less than 10^4^ /μL in 1 mL of sterile phosphate buffer solution (PBS, filtered with 0.22μm filter before use). Cell samples were analyzed at a flow rate of 10 μL/min until at least 10,000 cells were recorded. We chose this number because it could sufficiently and accurately characterize population cellular noise [11, 12]. Flow cytometry data (containing the standard deviation (*σ*) and mean (*μ*) of fluorescence intensity of each strain) were acquired with CytExpert software v2.3. Cells labeled with either GFP or BFP can be directly separated as shown in Fig. S2c.

*Quantitative real-time PCR (qPCR)* The absolute abundance of each strain was determined using qPCR according to the unique barcode within the genome of each mutant strain [1, 13, 14]. The frozen samples were heated at 98℃ for 5 minutes to obtain dissolved DNA. qPCR was performed in 20 μL reactions with 10 μL of SYBR Premix Ex Taq (Bio-Rad Laboratories, Inc.), 1 μL of DNA template, 2 μL of primer pairs and 8 μL of ddH_2_O. All samples were amplified and measured using a CFX Connect™ Real-Time System (Bio-Rad Laboratories, Inc., CFX Connect). The thermocycling protocol was based on manufacturer's specifications (initial denaturation at 95°C for 3 min, followed by 40 cycles of denaturation at 95°C for 5s, combined annealing and extension at 60°C for 30 s). Each of strain-specific amplicons was amplified from recombinant plasmids by PCR. To obtain the exact abundance of each strain, DNA templates with known abundance were used to generate standard curves, with 10-fold serial dilution. Given that all amplicons were single copied, the copy numbers can approximately represent the abundance of each of corresponding strains. qPCR test was performed based on three technical replicates of each biological replication. Primers used for qPCR are listed in Table S5.

*Amino acids quantification* Samples of *ΔargA-ΔmetJ* or *ΔmetA-ΔargR* under mono-culture taken at 8 HAI were first centrifuged at 8000 r.p.m. for 10 min. The supernatant was then filtered using 0.22 μm sterile filter membrane. The concentration of AAs in the filtrate was quantified using liquid chromatography-mass spectrometry (LC-MS, Agilent Technologies Inc., Agilent 6460) [1].

**Data analyses**

Data from Experiment 1 were further analyzed to quantify the magnitude of the cross-feeding effect on cell noise of each strain. This was achieved by decomposing the total cell noise of a strain in the cooperative consortium ($\eta_{comm}$) into two components: noise inherent from the population ($\eta_{pop}$) and noise induced by cross-feeding ($\eta_{cross-feeding}$), according to the following relationship: $\eta_{comm}^{2}= \eta_{pop}^{2}+ \eta_{cross-feeding}^{2}$.

Data from Experiment 2 were subject to linear model analysis. Data from Experiments 5 and 6 and AA production were subject to two-way ANOVA, followed with Tukey's HSD tests for multiple comparisons between pairwise groups. Most other data pertaining to cell noise were subject to two-sides *t*-test. All statistical analyses were performed using the *R* software (version 4.3, The R Foundation for Statistical Computing, 2023). Figures and/or graphs were prepared using the GraphPad Prism software (version 8, GraphPad Software). The contribution of cross-feeding to cell noise (in experiment 1) and the fold-change in cell noise (in experiment 8) were calculated using a bootstrap method. Statistically significant differences were accepted at *P* < 0.05.

*Bootstrap resampling method* First, we generated a bootstrap sample from the mono‑culture group by randomly drawing three values with replacement from cell noise measurements of three biological replicates. A corresponding bootstrap sample was generated simultaneously from the co‑culture group in the same way. Second, the target statistic was calculated from these two bootstrap samples. Third, this process was repeated 1,000 times to obtain a bootstrap distribution of the statistic. Fourth, the 50th percentile of the bootstrap distribution was taken as the point estimate, and the 2.5th and 97.5th percentiles as the 95% confidence interval.

2. **Supplementary notes**

**Supplementary note I**

The opposite trends for two strains expressing GFP and BFP are due probably to the combination effect of protein features (e.g. protein structure and degradation rate) and the number of codons in a given amino acid (arg or met) in the protein. The GFP sequence contains only 2.5% (6/238) arg codons, which is much lower than the percentage in the genome (5.8%, also see Table S1). Under low arg conditions, GFP synthesis requires fewer arg molecules than most endogenous genes, giving its competitive advantage, and it will result in a higher fluorescence intensity. As arg concentration increases, however, this advantage diminishes, and GFP fluorescence decreases (Fig. 2c). Such pattern holds true for GFP+3R and GFP+5R (Fig. 3c). For the met gradient, GFP contains about 2.5% (6/238) met codons, close to the genomic average of 2.8%, and its fluorescence increases with met concentration (Table S1 and Fig. S3j). Similar logic applies to BFP in both amino acid gradients (Fig. 2d and S3i); the difference between GFP and BFP might also be affected by protein-specific properties, such as protein stability. In sum, the overall pattern may differ because of codon composition and protein-specific properties, but it did not affect the cross-feeding effect on cell noise in the synthetic systems.

**Supplementary note II**

To theoretically assess whether nutrient heterogeneity exists in shaking media, we calculated the Damköhler number ($Da$) of the microbial cross-feeding system. The Damköhler number, defined as the ratio of the macroscopic mixing time to the reaction consumption time, is a measure to determine whether nutrient diffusion or consumption is the rate‑limiting step in a dynamic system. The equation for Damköhler number is given as:

$Da=\frac{T_{mix}}{T_{consumption}}$	(1)

where $T_{\text{mix}}$ is the characteristic time for a newly secreted amino acid molecule to be spread throughout the whole liquid volume, and $T_{consumption}$ is the characteristic time for consuming the amino acid by a recipient bacterium.

- If $T_{\text{mix}}\ll T_{consumption}$ ($Da\ll1$), mixing (diffusion) is much faster than consumption, suggesting that the amino acid is rapidly distributed within the bulk system before being consumed, and thus the whole system can be considered homogeneous.
- Conversely, if $T_{\text{mix}}\gg T_{consumption}$ ($Da\gg1$), consumption is far quicker than mixing; the produced amino acid molecule is quickly captured by the nearby recipients before it can be transported to a distant place, creating a spatial concentration gradient.

We then derived a quantitative relationship between the bulk concentration of AA and the mixing/reaction time by considering a steady‑state mass balance at a representative volumetric unit. Similar to the continuous stirred tank reactor (CSTR) model [15, 16], the net accumulation is zero at steady state, and the equation is expressed as:

$0=QC_{in}-QC_{bulk}-Vk_{consumption}C_{bulk}$	(2)

where $C_{\text{in}}$ represents the input concentration of the nutrient into the system,$C_{\text{bulk}}$ represents the background nutrient concentration in the system, *Q* denotes the flow rate of continuous feeding, *V* is the total volume of the system and $k_{consumption}$ is the consumption rate of AA.

- In our experimental system, $C_{\text{in}}$ is the concentration if mixing were infinitely fast and can be estimated using the value of the equivalent amino acid concentration supplied from cross-feeding consortia (i.e., 20 μM), $C_{\text{bulk}}$ can be estimated as the measured concentration of free AA in the bulk solution (i.e., 1 μM) (Table S2).
- $QC_{\text{in}}$ represents the total input rate of AA, with most of them from secretion and a small fraction diffused from the surrounding environment.
- $QC_{\text{bulk}}$ denotes the removal of AA from the bulk solution by convective/diffusive exchange.
- $Vk_{consumption}C_{\text{bulk}}$ is the volumetric consumption rate, with the first‑order rate constant $k_{consumption}=1/T_{consumption}$.

Define the mixing rate constant $k_{\text{mix}}=Q/V=1/T_{\text{mix}}$, then by dividing both sides of Equation (2) with the system volume $V$, we obtain:

$k_{mix}C_{in}-k_{mix}C_{bulk}-k_{consumption}C_{bulk}=0$	(3)

Rearranging Equation (3) gives the relationship between the bulk concentration and the input concentration:

$\frac{C_{bulk}}{C_{in}}=\frac{1}{20}=\frac{k_{mix}}{k_{mix}+k_{consumption}}=\frac{T_{consumption}}{T_{mix}+T_{consumption}}$	(4)

Substituting the quantitative relations (i.e., $T_{mix}$ and $T_{consumption}$) into the Damköhler number formula yields:

$Da=\frac{T_{\text{mix}}}{T_{consumption}}\approx19\gg1$	(5)

Though part of these parameters is simplified in equation (2), for example, the effective concentration of amino acid obtained by recipient cells might be higher than $C_{bulk}$ in reality, this simplification does not alter the qualitative trend.

The above derivations indicate that AA (e.g. arginine) secreted by an overproduced cell is largely taken up by a neighboring or nearby autotroph before it can be fully dispersed throughout the medium, resulting in a very low concentration of AA in relatively distant places. These theoretical derivations are also in agreement with our experimental observation that the concentrations of both amino acids in the bulk solution of the cross-feeding system were, due mainly to high consumption rate, extremely low (Table S2). All these derivations indicate that the amino acid distribution in the culture tube remains heterogeneous, even under shaking conditions.

**3. Supplementary Figures**

**Figure S1. Construction of a cooperative microbial consortium. a**. Schematic description of the metabolic cross-feeding system consisting of two engineered strains. The two strains, *ΔargA-ΔmetJ* (marked with a GFP, green fluorescent protein) and *ΔmetA-ΔargR* (marked with a BFP, blue fluorescent protein), cooperated by exchanging two essential amino acids (Met for methionine; Arg for arginine). **b-d**. Mono- and co-culture experiments. Growth of two strains in mono-culture without any amino acid (AA) addition (**b**), mono-culture amended with required AA (**c**) and co-culture (**d**). Data represent mean ± SEM (n = 3). **e-f**. GFP and BFP plasmid construction. Recombinant plasmids are constructed by inserting the gene sequences encoding EGFP (**e**) and mTagBFP2 (**f**) into the pET-3a plasmid. Ampicillin resistance gene (*ampR*) is constitutively expressed, and the fluorescent protein gene is regulated by the Lac promoter which was induced by IPTG. **g**. Effect of recombinant plasmids on the growth of *E. coli*. The growth of the original mutant without plasmid (mutant) and the mutants carrying GFP or BFP was quantified at 24 HAI. Data represent mean ± SEM (n = 3). **h**. Single-cell fluorescence intensity measurement. The value of cell fluorescence of each strain was observed using a flow cytometry. The scatter plots were generated by using FITC measurements as the x-axis and PB450 measurements as the y-axis, with each data point representing an individual cell. Three distinct clusters of cells are identified, with blue and green points representing bacteria expressing BFP and GFP, respectively, while black points denoting non-expressing bacteria (negative control). This panel is only for illustrating three populations with distinct fluorescence characteristics. The proportion of non‑fluorescent cells in the cooperative consortium was less than 5% (see Fig. 1a).

**Figure S2. Effect of cross-feeding on cell noise in synthetic microbial consortia.** **a-b**. Cross-feeding enhanced cell noise in the synthetic consortium consisting of strains *ΔargAΔmetJ* (**a**) and *ΔmetAΔargR* (**b**) over time. The two strains were growing under mono-culture (light colors, solid lines) and co-culture (dark colors, dashed lines) conditions over 48 hours (abbreviated as *mono* and *co* in the figure). **c-d**. Relationship between standard deviation (σ) and mean fluorescence intensity (μ) in mono‑culture and co‑culture of strains *ΔargAΔmetJ*-GFP (**c**) and *ΔmetAΔargR*-BFP (**d**). The standard deviation (σ) is plotted against the mean fluorescence intensity (μ), from 8 h to 48 h, and linear regression was performed for mono‑ and co‑culture, separately. Data from panels **a** and **b**, and each dot represents one biological replicate. *** indicates significance at *P* < 0.001, test for difference between slopes with dummy variable regression. **e-l.** Increased cell noise by cross-feeding in two synthetic consortia with different paired strains. **e-h**. “Arg‑Met” cross-feeding between *ΔmetAΔargR*-GFP and *ΔargAΔmetJ*-BFP. **e**. Schematic design of cross‑feeding of Arg and Met between the two strains. **f-g.** Growth curves of the two strains in mono‑culture (**f**) and co-culture (**g**). **h.** Cell noise of strains *ΔmetAΔargR*-GFP (green bars) and *ΔargA-ΔmetJ*-BFP (blue bars) under mono-culture or co-culture at 24 hours after incubation (HAI). **i-l**. “Arg‑Trp” cross-feeding between *ΔargAΔtrpR*-GFP and *ΔtrpCΔargR*-BFP. **i**. Schematic design of cross‑feeding of Arg and Met between the two strains. *ΔargA-ΔtrpR*, colored in light green. *ΔtrpC-ΔargR*, colored in yellow. **j-k.** Growth curves of the two strains in mono‑culture (**j**) and co-culture (**k**). **l.** Cell noise of strains *ΔargA-ΔtrpR* (green bars) and *ΔtrpC-ΔargR* (yellow bars) under mono-culture or co-culture at 24 HAI. **m-p**. Commensalism promotes unidirectional increase in cell noise. **m** and **o**. Schematic diagrams of synthetic consortia with commensalism. Commensalism‑1 (**m)**, *ΔargAΔmetJ* supplies methionine to *ΔmetAΔargR* in the presence of arginine (Arg). Commensalism‑2 (**o**), *ΔmetAΔargR* supplies arginine to *ΔargAΔmetJ* in the presence of methionine (Met). **n** and **p**. Cell noise of the two strains under mono‑ and co‑culture for two synthetic consortia with commensalism. All data represent mean ± SEM (n = 3). For panels **a** and **b**, significant differences (*P* < 0.05, two-tailed *t* test) between mono-culture and co-culture were observed at each time point for each strain, except for *ΔmetAΔargR* at 4 HAI. For panels **h**, **l**, **n** and **p**, *, *** and ns indicate significance at *P* < 0.05, *P* < 0.001 and *P* > 0.05, respectively (two-tailed *t* test).

**Figure S3. The growth, fluorescence intensity and cell noise of cooperative strains supplied with a gradient concentration of AAs. a-b.** Growth curves of *ΔargAΔmetJ* (**a**) and *ΔmetAΔargR* (**b**) over 24 hours in mono-culture (blue) supplied correspondingly with a gradient concentration of Arg and Met (5-30 µM) or in co-culture without any AA supply (red). **c-d.** Growth of *ΔargAΔmetJ* (**c**) and *ΔmetAΔargR* (**d**) in mono-cultures supplied correspondingly with a gradient concentration of Arg and Met (0, 10, 20, 30, 40, 50, 75 and 100 μM of Arg or Met). At 24 HAI, strain growth was measured using absorbance at 600 nm. **e-h**. Cell noise of strains *ΔargAΔmetJ* (**e**, **g**) and *ΔmetAΔargR* (**f**, **h**) incubated in mono-culture under different concentrations of Arg or Met. For panels **e** and **f**, concentrations ranged from 10 to 100 μM (data from Fig. 2**a**, 2**b**); for panels **G** and **h**, a denser set of low concentrations from 2 to 100 μM was used (2, 4, 6, 8, 10, 15, 20, 25, 30, 35, 40, 50, 100). Different letters denote statistically significant differences (One-way ANOVA with Tukey’s post-hoc test, *P* < 0.05). **i-j.** Relationship between standard deviation (*σ*) and mean (*μ*) of fluorescence intensities in different treatment groups (AA addition levels) of populations *ΔargAΔmetJ*-GFP (**i**) and *ΔmetAΔargR*-BFP (**J**). Each point represents a biological replicate from experiment 2 (also see Fig. 2**a**, 2**b**). **k-n.** Cell noise and fluorescence intensity of *ΔargAΔmetJ* and *ΔmetAΔargR* with exchanged fluorescent proteins (*ΔargAΔmetJ*-BFP and *ΔmetAΔargR*-BFP) under the supply of a gradient concentration of corresponded AA. Cell noise (**k, l**) remained unchanged (slopes: −0.021% ± 0.046% and −0.044% ± 0.056%), while the fluorescence intensity (**m, n**) increased linearly (slopes: 108 ± 30 and 200 ± 39) with increasing the concentrations of AAs. R² and *P* values are shown in each of panels. All data represent mean ± SEM (n = 3).

**Figure S4. Proposed mechanism for the effect of cross-feeding on cell noise in the metabolic cross-feeding system. a.** In mono-culture with uniform external nutrient supply, amino acids (AAs) are homogeneously distributed. This results in low cell-to-cell variation in intracellular AA pools, leading to synchronized gene expression and low population noise. **b.** In co-culture with metabolic cross-feeding, AAs are primarily derived from stochastic secretion by partner cells, creating a spatially heterogeneous nutrient landscape. The concentrations of AA surrounding individual cells vary substantially, likely resulting in enhanced variation in gene expression and subsequent population-wide cell noise.

**Figure S5. Validation of GFP mutants. a.** Amplification of cell noise of strain *ΔmetAΔargR*-BFP in three synthetic consortia, with the co-culture including *ΔargA-ΔmetJ* carrying GFP mutants (with 0, 3 or 5 arginine codons), respectively. **b**. Cell noise of strain *ΔmetAΔargR* carrying GFP mutants in mono- and co-culture. Data represent mean ± SEM (n = 6 in panel a, n = 3 in panel b). *** denotes statistical significance at *P* < 0.001 (two-tailed *t* test).

**Figure S6. Spatial, but not temporal, nutrient heterogeneity increases cell noise.** **a** and **b.** Heterogeneous distribution of strains in culture tubes with the initial bacterial density (OD) being set at 0.6 (**a**) or 0.3 (**b**). The data for OD set at 1 are shown in Figure 4a. Each dot represents an individual sampling point from either the edge or the center of the culture tube under two conditions: shaking at 200 rpm or mixing with a thorough vortex. The variance in strain abundances between shaking and vortex conditions (each consisting of 5 replicates × 2 positions = 10 samples) was compared using an *F*‑test (***, *P* < 0.001). **c-e**. Effect of temporal nutrient heterogeneity on cell noise of two strain populations in mono-culture. **c**. Schematic description of the experiment design for simulating temporal resource heterogeneity. Two temporal nutrient heterogeneity treatments were set: (i) One-shot addition (30 µM total), simulating an initial excess of AA that remained in excess throughout the 12-hour cultivation despite consumption; (ii) Hourly addition (2.5 µM per hour), simulating an oscillating nutrient concentration, due to the cycle of supplementation and consumption. Both *ΔargA-ΔmetJ* and strain *ΔmetA-ΔargR*, supplied respectively with Arg and Met, were growing in monoculture over 12 hours. **d** and **e**. Changes in cell noise of *ΔargA-ΔmetJ* (**d**) and *ΔmetA-ΔargR* (**e**) over time. Solid dots, one-shot nutrient addition treatment. Hollow dots, hourly nutrient addition treatment. Data represent mean ± SEM (n = 3).

**Figure S7. The growth and cell noise of *E. coli* co-cultured with bacterial strains isolated from different environments. a-c**. Growth curves of the engineered *E. coli* (colored in green) and three environmental isolates incubated in mono-culture (dashed lines) or co-culture (solid lines). The three environmental strains A1 (**a**, colored in blue), S1 (**b**, colored in blue) and R1 (**c**, colored in blue) were isolated from aquatic, bulk soil and plant rhizospheric environments. The growth of *E. coli* was stimulated by all three environmental isolates. The presence of *E. coli* facilitated the growth of A1 and S1 but not R1 over the incubation time. **d-f**. The growth and cell noise of *E. coli* in synthetic microbial consortia with negative or transitional interactions over time. The three environmental strains A2 (**d**), S2 (**e**) and R2 (**f**) were isolated from aquatic, forest soil and corn rhizospheric environments as well (also see the cited ref. [17]). The fold-changes in cell noise of *E. coli* are shown in the left y axis (red or blue bars), while the growth curves are presented in the right y axis (grey lines). Dashed and solid grey lines denote the abundance of *E. coli* in mono-culture or co-culture, respectively. Red bars indicate an increase in cell noise, while blue bars represent a reduction in cell noise of *E. coli* populations. The change of cell noise was calculated as follows: $Noise change ={(\eta_{comm}-\eta_{pop})}/{\eta_{pop}\times100\%}$, where $\text{η}_{\text{comm}}$ and $\text{η}_{\text{pop}}$ represent cell noise in co-culture (community level) and mono-culture (population level), respectively. Noise change was assessed by bootstrap resampling and indicated with median values accompanied by upper and lower quartile markers. All data represent mean ± SEM (n=3).

**Figure S8. Community composition over time in the evolution experiment. a-c.** The flow-cytometric profiles of *E. coli* evolving in mono-culture, recorded on day 10 (**a**), day 20 (**b**), and day 30 (**c**). **d-j.** The flow-cytometric profiles of the three evolutionary lines of cocultured *E. coli* (colored in green) and A1 (colored in black). **d-f**, line 1. **g-i**, line 2. **j**, line 3. Note that evolutionary line 3 collapsed on day 10 following the extinction of strain A1. Data are derived from Experiment 8. FSC-A (forward scatter-area) indicates cell size, while SSC-A (side scatter-area) reflects internal complexity. Their combined measurement enables distinction of different cell populations in heterogeneous samples.

**Figure S9. A conceptual framework for the effect of cross-feeding on cell noise in microbial communities.** Metabolic cross-feeding, which could be extended to those with positive interactions including commensalism and exploitation, will beget resource (e.g. metabolic nutrients) heterogeneity within a microbial community. Increased resource heterogeneity, which can be also driven by uneven distribution of cooperative strains and/or heterogeneous spatial structure, will induce cell-to-cell variation in gene expression, resulting in an increase in cell noise of microbial populations in the cooperative community. Amplification of cell noise may negatively feed back to the cooperative system, raising questions about the long-term stability and maintenance of positive interactions in microbiomes in nature. +, positive effect. - negative feedback.

**Figure S10.** **Effects of competition on cell noise. a** and **c.** Schematic diagrams of synthetic microbial consortia with competition (-/-). **a**. Competition‑1 consortium: *ΔargAΔmetJ* and *ΔmetAΔargR* co‑cultured in minimal medium supplemented with both arginine (Arg) and methionine (Met). **c**. Competition‑2 consortium: *ΔargAΔmetJ* and *ΔargAΔtrpR* (tryptophan‑overproducing) co‑cultured in minimal medium supplemented with arginine (Arg). **b** and **d.** Cell noise of the two strains under mono‑culture (mono) and co‑culture (co) in the two synthetic consortia. “ns” indicates *P* > 0.05 (*t*-test). Data represent mean ± SEM (n=3).

**4. Supplementary Tables**

**Table S1. Percentage of Arg and Met in gfp, bfp genes or in the genome.**

| **Genes** | **Amount of Arg** | **Proportion of Arg (%)^†^** |
| --- | --- | --- |
| gfp (gfp+0R) | 6 | 2.5 |
| gfp+3R | 9 | 3.8 |
| gfp+5R | 11 | 4.6 |
| bfp | 8 | 3.4 |
| genome^††^ |  | 5.8 ± 2.2 |
| **Genes** | **Amount of Met** | **Proportion of Met (%)^†^** |
| gfp | 6 | 2.5 |
| bfp | 8 | 3.4 |
| genome^††^ |  | 2.8 ± 1.2 |

† This value represents the percentage of arginine or methionine in the codon sequence of specific genes.

†† This value represents the average amino acid percentage of all genes on *E. coli* genome. This data is from [18], and represents as Mean ± SD.

**Table S2. The concentrations of free** **AAs in co-culture.**

| **Samples** | **replicate 1****^†^** | **replicate 2^†^** |
| --- | --- | --- |
| Arg-12h | 8.28 ng/mL （0.048 μM） | 7.82 ng/mL（0.045 μM） |
| Met-12h | 114 ng/mL（0.764 μM） | 116 ng/mL（0.777 μM） |
| Arg-24h | 1. 49 ng/mL（0.009 μM） | 1.86 ng/mL（0.011 μM） |
| Met-24h | 75.7 ng/mL（0.507 μM） | 75.9 ng/mL（0.509 μM） |

**†** The concentration of AAs in supernatant liquor in co-culture of strain *ΔargAΔmetJ* and *ΔmetAΔargR* is determined by LC-MS.

**Table S3. Strains and plasmids used in this study.**

| **Strain or plasmid** | **Description** | **Source** |
| --- | --- | --- |
| ***E. coli* Strain** |  |  |
| MG1655 | Wild-type | Lab stock |
| *ΔargA-ΔtrpR* | Arg auxotroph and Met overproducer | This study |
| *ΔtrpC-ΔargR* | Trp auxotroph and Arg overproducer | This study |
| *ΔargA-ΔmetJ* | Arg auxotroph and Met overproducer | This study |
| *ΔmetA-ΔargR* | Met auxotroph and Arg overproducer | This study |
| BL21-GFP | *E. coli* strain BL21 constitutively expressed GFP | This study |
| **Isolated strain** |  |  |
| **A1** | *Priestia megaterium* strian F20 | Isolated from lake & Lab stock |
| **A2** | *Priestia megaterium* strian B13 | Isolated from lake & Lab stock |
| **S1** | *Mesorhizobium* sp. strian L-8-3 | Isolated from soil & Lab stock |
| **S2** | *Arthrobacter* sp. strian YN | Isolated from soil & Lab stock |
| **R1** | *Brevundimonas diminuta* strian KX-1 | Isolated from rhizosphere & Lab stock |
| **R2** | *Klebsiella quasipneumoniae* strian G4584 | Isolated from rhizosphere & Lab stock |
| **Plasmid** |  |  |
| pBFP | Plasmid encoding BFP; Amp^r^ | This study |
| pGFP | Plasmid encoding original GFP (GFP+0R); Amp^r^ | This study |
| pGFP+3R | Plasmid encoding GFP+3R; Amp^r^ | This study |
| pGFP+5R | Plasmid encoding GFP+5R; Amp^r^ | This study |

**Table S4. Primers used for the construction of mutants.**

| **Primer** | **Sequence^†^（5’-3’）** |
| --- | --- |
| *argA*-5O | GGGGACAAGTTTGTACAAAAAAGCAGGCTGCGAACTGCTTATATTTCAGC |
| *argA*-5I | ACTATATGTGAAGGCATGGCAACTCGGTTTTACGTTCCTTT |
| *argA*-3I | GCCATGCCTTCACATATAGTGTTCATCAAGGGGTGAAGTT |
| *argA*-3O | GGGGACCACTTTGTACAAGAAAGCTGGGTGGCGATGACGGTACATAAAT |
| *argR*-5O | GGGGACAAGTTTGTACAAAAAAGCAGGCTCATTTAATTGCCGTAATCAGG |
| *argR*-5I | GAGTGATCCATACACATTCCTCGCATAAGTCACCCGATA |
| *argR*-3I | GGAATGTGTATGGATCACTCGGAGCTTTAATCTCTGCCC |
| *argR*-3O | GGGGACCACTTTGTACAAGAAAGCTGGGTCGCGCTAACTGTTTCAGTT |
| *metA*-5O | GGGGACAAGTTTGTACAAAAAAGCAGGCTCCACTTAGCAACATAAATCCAA |
| *metA*-5I | TAGCACACGGTCGAATTTCCACACGAATCGGCATAACC |
| *metA*-3I | GGAAATTCGACCGTGTGCTACCAACTGGCTCAACTATTACG |
| *metA*-3O | GGGGACCACTTTGTACAAGAAAGCTGGGTGAAGGATCGTTGTGCTCTTT |
| *metJ*-5O | GGGGACAAGTTTGTACAAAAAAGCAGGCTGTCAGGCACCAGAGTAAACAT |
| *metJ*-5I | GATACGCGCCAATCTCTATACTCCATTCAGCCATGAGATAC |
| *metJ*-3I | TATAGAGATTGGCGCGTATCCGTGGGAATACTAAAGCAAAA |
| *metJ*-3O | GGGGACCACTTTGTACAAGAAAGCTGGGTAAGGCAGGTACAGAAACCAG |
| *trpC*-5O | GGGGACAAGTTTGTACAAAAAAGCAGGCTTGATATGAACGCCGATAAAT |
| *trpC*-5I | GCACATCTGACAACAGGCGAACGGTTTGCATCATTTACC |
| *trpC*-3I | TCGCCTGTTGTCAGATGTGCGCGCATATTAAGGAAAGGA |
| *trpC*-3O | GGGGACCACTTTGTACAAGAAAGCTGGGTATGGTCGGATAAGGATGC |
| *trpR*-5O | GGGGACAAGTTTGTACAAAAAAGCAGGCTGTTACTGATCCGCACGTTT |
| *trpR*-5I | GCGCAGAAGGCAATGCTATAGATTGTTGGGCCATAATATGT |
| *trpR*-3I | TATAGCATTGCCTTCTGCGCTGAAAAGCGATTGATTTTGTA |
| *trpR*-3O | GGGGACCACTTTGTACAAGAAAGCTGGGTTATTTGTAGGACGGATAAGGC |
| *menF*-5O | GGGGACAAGTTTGTACAAAAAAGCAGGCTATCCCTGTGACGGAGGTCTA |
| *menF*-5I | TATTAATTTCGCGGGATCGACCTCTCCCTGTTAAAAGCC |
| *menF*-3I | GATGAACTATACAAATAATAATGAGTCGCATCATTACC |
| *menF*-3O | GGGGACCACTTTGTACAAGAAAGCTGGGTCCGGATAGAGATTTGCCACC |

**†** Specific homology arm sequences of each gene are underlined in black, and unique genomic barcodes (used for qPCR identification) are underlined in red.

**Table S5. qPCR primers used for quantifying each mutant.**

| **Primer** | **Sequence (5’-3’)** |
| --- | --- |
| *argA*-F | GGCATTACTGATAAAAAAGTCGC |
| *argA*-R | ACACTATATGTGAAGGCATGGC |
| *argR*-F | GGCATGTTTCTCAATAACGAAA |
| *argR*-R | CGAGTGATCCATACACATTCCT |
| *essD*-F**^†^** | ATGGCACCTCAGCAGGTAGT |
| *essD*-R**^†^** | CAGCCTTGCGCTTATCTTCT |
| A1-*alsS*-F | AGCTCTCGTGAAACAGCTCC |
| A1-*alsS*-R | GCTTGCCGCAGGATGTAATG |
| S1-*phnG*-F | CAGTCCTCTCCGCGTACCAT |
| S1-*phnG*-R | CGGTCATGCCTATGCTCTCG |
| R1-*mltA*-F | GGCGAGGCCAATAAAAAGCC |
| R1-*mltA*-R | TGTCACCATTTGCGGGATGA |

**†** Primer pair *essD*-F and *essD*-R were used to measure the abundance of *E. coli* in experiment 7.

**5. Cited references**

1.	S. Li *et al.* Synthetic microbial consortia with programmable ecological interactions. *Methods in Ecology and Evolution* 2022*;* **13**: 1608-1621.

2.	M. Jin *et al.* Unique organizational and functional features of the cytochrome *c* maturation system in *Shewanella oneidensis*. *PLoS One* 2013*;* **8**: e75610.

3.	F. W. Studier, B. A. Moffatt. Use of bacteriophage T7 RNA polymerase to direct selective high-level expression of cloned genes. *Journal of Molecular Biology* 1986*;* **189**: 113-130.

4.	B. Kleeman *et al.* A guide to choosing fluorescent protein combinations for flow cytometric analysis based on spectral overlap. *Cytometry Part A* 2018*;* **93**: 556-562.

5.	M. T. Mee *et al.* Syntrophic exchange in synthetic microbial communities. *Proceedings of the National Academy of Sciences of the United States of America* 2014*;* **111**: 2149-2156.

6.	A. Urchueguía *et al.* Genome-wide gene expression noise in Escherichia coli is condition-dependent and determined by propagation of noise through the regulatory network. *PLoS Biology* 2021*;* **19**: e3001491.

7.	R. E. Lenski. Revisiting the Design of the Long-Term Evolution Experiment with Escherichia coli. *Journal of Molecular Evolution* 2023*;* **91**: 241-253.

8.	P. M. Doran, *Bioprocess Engineering Principles, Edition 2*. (Academic Press, 2012).

9.	M. B. Elowitz *et al.* Stochastic gene expression in a single cell. *Science* 2002*;* **297**: 1183-1186.

10.	J. M. Raser, E. K. O'Shea. Noise in gene expression: Origins, consequences, and control. *Science* 2005*;* **309**: 2010-2013.

11.	E. M. Ozbudak *et al.* Regulation of noise in the expression of a single gene. *Nature Genetics* 2002*;* **31**: 69-73.

12.	W. J. Blake *et al.* Noise in eukaryotic gene expression. *Nature* 2003*;* **422**: 633-637.

13.	C. Xu *et al.* Coupled anaerobic methane oxidation and metal reduction in soil under elevated CO_2_. *Global Change Biology* 2023*;* **29**: 4670-4685.

14.	K. Zhang *et al.* Inhibition of autotrophic nitrifiers in a nitrogen-rich paddy soil by elevated CO_2_. *Nature Geoscience* 2024*;* **17**: 1254–1260.

15.	F. Carolin Christopher *et al.* A review on critical assessment of advanced bioreactor options for sustainable hydrogen production. *International Journal of Hydrogen Energy* 2021*;* **46**: 7113-7136.

16.	P. Safaeian *et al.* P3HB from CH4 using methanotrophs: aspects of bioreactor, fermentation process and modelling for cost-effective biopolymer production. *Frontiers in Bioengineering and Biotechnology* 2023*;* **11**: 1137749.

17.	T. Sun, PhD Thesis, Interspecific interactions shape cell noise in synthetic microbial communities (In Chinese). Zhejiang University, Hangzhou (2025).

18.	J. R. Lobry, C. Gautier. Hydrophobicity, expressivity and aromaticity are the major trends of amino-acid usage in 999 *Escherichia coli* chromosome-encoded genes. *Nucleic Acids Research* 1994*;* **22**: 3174-3180.
